# Engineered metal based nanoparticles and innate immunity

**DOI:** 10.1186/s12948-015-0020-1

**Published:** 2015-07-15

**Authors:** Claudia Petrarca, Emanuela Clemente, Valentina Amato, Paola Pedata, Enrico Sabbioni, Giovanni Bernardini, Ivo Iavicoli, Sara Cortese, Qiao Niu, Takemi Otsuki, Roberto Paganelli, Mario Di Gioacchino

**Affiliations:** Immunotoxicology and Allergy Unit, Ageing Research Center G. d’Annunzio University Foundation, Chieti, Italy; Department of Medicine and Science of Ageing, G. d’Annunzio University, Chieti, Italy; Occupational Medicine, II University, Naples, Italy; Department of Biotechnology and Molecular Biology, University of Insubria, Varese, Italy; ‘Protein Factory’, Interuniversity Center of the Politecnico di Milano and University of Insubria, Milan, Italy; Institute of Public Health, Catholic University of the Sacred Heart, Rome, Italy; School of Public Health, Shanxi Medical University, Taiyuan, People’s Republic of China; Department of Hygiene, Kawasaki Medical School, Kurashiki, Okayama 7010192 Japan

**Keywords:** Immunotoxicity, Cell receptors, Immunostimulation, Barriers, Adjuvant, Cytokines

## Abstract

Almost all people in developed countries are exposed to metal nanoparticles (MeNPs) that are used in a large number of applications including medical (for diagnostic and therapeutic purposes). Once inside the body, absorbed by inhalation, contact, ingestion and injection, MeNPs can translocate to tissues and, as any foreign substance, are likely to encounter the innate immunity system that represent a non-specific first line of defense against potential threats to the host. In this review, we will discuss the possible effects of MeNPs on various components of the innate immunity (both specific cells and barriers). Most important is that there are no reports of immune diseases induced by MeNPs exposure: we are operating in a safe area. However, in vitro assays show that MeNPs have some effects on innate immunity, the main being toxicity (both cyto- and genotoxicity) and interference with the activity of various cells through modification of membrane receptors, gene expression and cytokine production. Such effects can have both negative and positive relevant impacts on humans. On the one hand, people exposed to high levels of MeNPs, as workers of industries producing or applying MeNPs, should be monitored for possible health effects. On the other hand, understanding the modality of the effects on immune responses is essential to develop medical applications for MeNPs. Indeed, those MeNPs that are able to stimulate immune cells could be used to develop of new vaccines, promote immunity against tumors and suppress autoimmunity.

## Introduction

Human exposure to engineered nanoparticles (NPs) is at present widespread. The general population is subjected to a lower risk compared to workers of industries producing or using NPs [1] and, obviously, extremely relevant is the exposure of patients receiving NP-based drugs for diagnostic or therapeutic purposes. NPs are used as biosensors, photo-detectors, sensing devices, catalysts, sorbents, semiconductors [2]. In nanomedicine, they are used in diagnostic imaging, vaccines, cancer therapy and drug delivery [3–6]. In any way, either assimilated by environmental exposure or deliberately administered to humans, they may interact with cellular and molecular targets and can also trigger unpredicted and potentially harmful outcomes. Therefore, information on biological effects and safety of these emerging manufactured products is mandatory.

Speculations on the possible effects of metal-based nanoparticles (nanoparticles made of elemental metals and their oxides and compounds - MeNPs) on human cells is not straightforwardly deducible from bulk metallic matter. This is because in the nanoworld, dose and concentration are not the most relevant factors for the toxicological profile but, rather surface specific area and physicochemical properties, such as ions leakage, magnetism, crystalline and electronic configurations should be taken into account [7, 8].

Moreover, metal toxicology definition is further complicated by the biphasic dose–response relationship of some metallic elements, the so-called hormetic-like behavior [9]. For doses below the threshold, the response can likely reflect an adaption of modest magnitude [10]. Additional complexity is given by the aggregation/agglomeration tendency of certain types of MeNPs and the tunable effect of the milieu [11]. The fact that a large number of studies are published on technical more than biomedical journals is symptomatic of the difficulty to adapt current biological tests to the assessment of NP toxicology.

Once inside the body, absorbed by inhalation, contact, ingestion and injection, MeNPs can translocate to tissues and, as any foreign substance, are likely to encounter the innate immune system [12]. The first response of the innate immune system to harmful substances is mediated by inflammation. The process is initiated by cells present in all tissues mainly resident as macrophages, dendritic cells, neutrophils and mast cells. They produce a variety of chemical factors that induce vasodilation and favor chemotaxis of phagocytes [13]. Generating high levels of reactive oxygen species (ROS) upon interaction with cellular components [14] causes accumulation of oxidized glutathione (GSSG). The extent of the generated oxidative stress determines the pathophysiologic potential of the NPs as it activates pro-inflammatory signals, favoring cell death [14] or cancer [15].

Cells of the innate immune system, particularly macrophages and dendritic cells, communicate with the adaptive immune system inducing tolerance or an immune reaction depending from the safety or harmfulness of the absorbed substance [13].

Numerous experimental evidences support the role of the innate immune cells in the onset of inflammatory pathways in response to MeNP exposure. Immunotoxicity comprises activation/dysregulation of macrophages and antigen presenting cells (APC) [16, 17].

There are evidences that in animals, MeNPs cause aspecific immune responses, immunosuppression and autoimmunity and associated morphological alterations of the immunologically active tissues [18]. These effects are attributable to both physical characteristics (mainly size) of NPs and to their chemical nature (through released ions) [19–21]. Moreover, MeNPs may affect immune system function indirectly by changing essential elements homeostasis due to released ions [22]. Metallic elements, their ions and compounds, have been clearly related to immune system mediated diseases. The finding that exposure to platinum, chromium, nickel, beryllium, and mercury can cause asthma and allergic contact dermatitis in professional workers provided the first clue of their possible detrimental influence [23].

There is no information in literature regarding the correlation of the onset of some disease as consequence of MeNPs exposure in the general population. However, it has been observed the increase in palladium (Pd) allergic contact dermatitis, in parallel with the increased pollution of PdNPs emitted from catalytic converters [24]. The main concern is for people exposed to large amounts or high concentrations of MeNPs, as workers in NP-making industries or in patients receiving NP-based drugs.

## Review

In this review, we will examine the possible immune effects of MeNPs, focusing our attention on the innate immune system (both specific cells and barriers), and we will discuss future research lines.

### Nanoparticle/cell surface receptor interaction

Increasing experimental evidences suggest that cells of the innate immune system react to MeNPs through the same mechanisms developed to destroy pathogens. Toll-like receptors (TLRs) are crucial sensor molecules, which detect conserved molecular patterns of microbes and viruses and initiate innate immune response [25]. Toll-like receptor 4 (TRL-4) appears to be sensor and signal transducer for CoNPs ending up with activation of the innate immune response and pro-inflammatory cytokines production [26]. In fact, no pro-inflammatory response is produced when using blocking antibodies against TRL-4 or when TLR4-negative cells are challenged, whereas an enhanced reaction is observed by TLR4-overexpressing cells [26]. Through the activation of TRL-4, human monocyte/macrophages THP-1 exposed to Co microparticles (0.5-2 μm) activate a signaling pathway leading to IL-8 release. This presumably leads to neutrophil attraction that in turn might phagocytose and eliminate the particles. An analogous IL-8-mediated mechanism might function for aggregated/agglomerated CoNPs, on epithelial cell types [27–29].

Non-toxic exposure (10 μg/mL) to TiO_2_ NPs and ZnO NPs did not significantly alter the phenotype of MDDC, whereas subtoxic concentrations of ZnO NPs, but not of TiO_2_ NPs, induced a down regulation of Fcγ RIII (CD16) expression on NK-cells, suggesting an effect on FcγR-mediated immune responses [30].

Gold nanoparticles (AuNPs) of various sizes (ranging from 4 to 45 nm) have been found to affect macrophage response against microbial pathogens through accumulation in the lysosomes and inhibition of TLR-9 function. In fact, AuNPs impede the binding of bacterial DNA fragments (CpG-ODN) specifically recognized by this receptor, as well as the downstream signaling pathway involving JNK and NF-kB activation, leading to lower TNF-α production. AuNPs are easily internalized by phagocytic and tumor-infiltrating macrophages used as a sort of Trojan horse to target (silica-coated) Au-nanoshells to the center of human breast carcinoma spheroids, thus allowing the killing of bystander tumor cells [31]. Similarly, AuNPs are internalized by human T cells and targeted to the tumor in a xenograft model of lymphoma [32]. AuNPs, commonly considered biologically inactive and non-cytotoxic, have become one of the ideal nanomaterials for medical applications. However, once engulfed by phagocytes, the immunological effects of AuNPs are still of concern and require exhaustive investigation. Table 1 summarizes the main interactions of MeNPs with receptors of the innate immune cells.

**Table 1:** Main interactions of metal nanoparticles with receptors of the immune cells

NP	Cells	Effect	Dose/Diameter	Ref
CoNP	Monocyte/Macrophages	-Activation of the innate immune response		[26]
		-Release of inflammatory cytokines through TLR-4		
TiO_2_NP ZnONP	Human lymphocytes	-No alterations of MDDC phenotype	10 μg/mL	[30]
ZnONP	Human lymphocytes	-Down regulation of CD16 on NK-cells	subtoxic	[30]
AuNP	Macrophages	-Reduction of macrophage response against pathogens	4-45 nm	[32]
		-Accumulation of NPs in the lysosomes		
		-Inhibition of TRL9 function		
		-Inhibition of TNF-α production		

### Immune depression and pro-inflammatory response

Monocytes/macrophages exposed to MeNPs (Co-, ZnO-, CeO_2_- and TiO_2_NPs) can die by necrosis and apoptosis, a phenomenon that depends on concentration, chemical nature, size and structure of NPs [33–37] as assessed with various *in vitro* assays. At non-cytotoxic doses, however, MeNPs generate pro-inflammatory effects. AgNP-exposed macrophages were promptly induced to produce IL-8, as well as oxidative stress genes (hemeoxygenase-1, heat shock protein-70), in a size-dependent way. In fact, 5 nm NPs produced an early effect, while 100 nm particles failed to do so [38]. The inverse relationship between size and cytotoxic effect of AgNPs is confirmed on human blood monocytes that produce higher levels of hydrogen peroxide when exposed to 5 nm compared to 28 nm NPs. Moreover, the potential of activating the innate immunity, measured as production of IL-1β, and induction of inflammasome formation and other effects, was higher for the smaller AgNPs [39]. ZiONPs behave similarly. As for Ag, at an equivalent mass load, smaller particles induce a greater cytotoxicity in exposed monocyte/macrophages [40].

Interestingly, in human peripheral blood lympho/monocytes, CoNPs induced an increase of TNF-α and IFN-γ release along with an inhibition of IL-10 and IL-2, a cytokine pattern similar to that detected in the experimental and clinical autoimmunity and in allergic contact dermatitis [41]. It is not possible to exclude that this pro-inflammatory response could be triggered by Co^2+^ ions that are known to abundantly leak from CoNPs.

Many metal oxide NPs (made of CuO, TiO_2_, ZnO, Fe_2_O_3_, Fe_3_O_4_) induce cytotoxicity and DNA damage in A549 type II lung epithelial cells. Amongst them, CuNPs evoke inflammatory responses stronger than the other metal oxides [42], and in an in vivo model, they induced an increase of neutrophils and associated cytokines in the lung as well as signs of cytotoxicity [43]. However, when mice were exposed to both CuNPs and *Klebsiella*, bacterial clearance was decreased respect to mice not treated by NPs. This suggests that CuNPs exposure might lead to increased risk of pulmonary infection by impairing host defense against bacteria. Similar results have been obtained by other authors with single-walled carbon nanotube [44]. However, it is not certain whether these NPs directly impair the immune system. It has been demonstrated that the rate of bacterial clearance depends on the ratio of neutrophils to bacteria and that a reduced recruitment of neutrophils with a severe neutrophil inflammation are accompanied by a reduced bacterial killing [45, 46].

Intratracheally instilled Fe_2_O_3_NPs induce lung inflammation as assessed by increased cytokine productions by cultured lung lymph node cells and decreased pulmonary immune responses against sheep erythrocytes. Both levels of inflammation and immunodepression were greater than those induced by the corresponding microparticles [47].

A recent *in vitro* study enlightened the differential response of human antigen presenting cells with different roles in innate and adaptive immunity, macrophages and dendritic cells, to ZnO and TiO_2_ NPs. ZnONPs caused cell death in a dose-dependent manner, but at sub-toxic doses both kinds appear to follow the typical storage, transport and detoxification route of metals through upregulation of the gene encoding metallothioneins. Nevertheless, dendritic cells appear less distressed by ZnONPs compared to macrophages, with only 12 genes affected, compared to the 2703 genes in macrophages. In macrophages, ZnONPs affect main biological processes regarding cell death and growth and controlling the development of the immune system. This effect was essentially dependent on particle dissolution and was strongly reduced when NPs were modified to reduce Zn^2+^ release [48].

Heavy metals and almost all MeNPs can also activate autophagy [35, 49–52], a fundamental eukaryotic pathway controlling inflammation through regulatory interactions with innate immune cells, by removing endogenous oxidative stress-damaged mitochondria and modulating the secretion of immune mediators [53–55]. Moreover, autophagy contributes to antigen presentation and to T cell homeostasis, and it affects T cell repertoires and polarization [56].

Mitogen-stimulated human primary lympho/monocytes exposed to Co and PdNPs show autophagic vacuoles, associated with the alteration of cell cycle, in particular with prolongation of G1-phase [20, 24], and, in the case of hematopoietic progenitor cells, with prolongation of G2/M-phase [20]. Delayed cell cycle-phases are likely due to DNA replication fidelity checkpoints that, in case of failure, cause mutations and genomic arrangements promoting cancer development. Moreover, IL-8 was released by the primary human lympho/monocytes upon exposure to a sub-toxic dose of PdNPs. Notably, such effect was not observed for immature progenitor cells of the myeloid lineage (CD133+) [57].

Two studies on the divergent behavior of NPs from particles of greater size (microparticles) or ions [58, 59] show that CoNPs cytotoxicity is lower than that of microparticles and ions, following the ranking ions > micro > nano. On the contrary, only micro- and nanoparticles have morphological transforming potential [58]. Another investigation concerning the interference of CoNPs with gene expression has shown that only Rab18 is affected by all three forms of cobalt. This gene regulates membrane traffic and vesicular organization, whose down-regulation is implicated in lipid metabolism, autophagy and inflammation. In general, Co ions interfere with genes related to mitochondrial dysfunction, microparticles with genes related to cell metabolism and cycle, whereas NPs with genes involved in the activation of the immune response, in particular innate immunity and apoptosis [60, 15].

Primary dendritic cells were the least sensitive to Co ions amongst six cell lines of non-immune system derivation and the second least sensitive to CoNPs, based on the hypothesis that the toxic effects of aggregated CoNPs are mainly due to Co ions dissolution from the aggregated NPs [29].

At present, a major area of interest is the modification of some properties of NPs (size, surface charge, hydrophobicity/hydrophilicity, and the steric effects of particle coating) that can dictate nanoparticle compatibility with the immune system in order to use them for human therapy [61–64]. For example, the hydrophilic environment, obtained designing NPs by attaching to poly(ethylene glycol) (PEG) or other types of polymers, shields them from immune recognition [65]. However, also in this case repeated injections of high doses of PEG-coated liposomes are followed by the formation of PEG-specific antibodies [66, 67], which results in an accelerated clearance of PEG-liposomes with change in their pharmacokinetic profile [68].

Coating can reduce NPs cytotoxicity and pro-inflammatory effects. However, while Fe_2_O_3_NPs coated with PEG or dextran were non-toxic to primary human monocyte-derived macrophages, dose-dependent toxicity of 30 nm and 50 nm silica-coated Fe_2_O_3_NPs was observed for primary monocyte-derived dendritic cells [69]. Similarly, both coating and particle size determine the cytotoxicity of ZnONPs for human macrophages and monocytes [40]. Poly(vinylalcohol)-coated super-paramagnetic Fe_2_O_3_NPs, used in biomedical applications induce important functional deficit in monocyte-derived dendritic cells. These cells internalize NPs in a dose-dependent manner. However, LPS-induced maturation decreases uptake at higher particle concentrations, and cytochalasin D pre-treatment also inhibits this process suggesting pinocytosis mediated by actin assembly. NPs exposed dendritic cells maintain the typical immunophenotype (CD80, CD83, CD86, myeloid/plasmacytoid DC markers) and are capable of antigen-uptake. However, the capacity of antigen processing, T helper cells stimulation, and cytokines induction is reduced, suggesting that they may revert to an immature state following particle exposure [70].

The type of coating also affects NPs uptake; for instance, silica-coated Fe_2_O_3_NPs are internalized to a significantly higher degree when compared to the dextran-coated NPs of comparable size, through an active, actin cytoskeleton-dependent process [69]. This behavior makes them promising materials for medical imaging and cell tracking. Table 2 summarizes cytotoxic effects of MeNPs.

**Table 2:** Main cytotoxic effects of metal nanoparticles on cells of the immune system

NPs	Cells	Effects	Ref
CoNPs ZnONPs CeO_2_NPs TiO_2_NPs	Mononyte/Macrophages	Necrosis (dependent on dose, size, concentration, structure	[33–36, 48]
AgNP ZiONP	Monocyte/Macrophages	-IL-8 and IL-1β production (size-dependent)	[37–40]
		-Oxidative stress	
		-Inflammasome induction	
CoNPs	Human PBMCs	-Increase of TNF-α, IFN-γ	[41]
		-Inhibition of IL-2, IL-10	
CuONP	Neutrophils	-Neutrophil recruitment and activation	[42, 43]
		-Increase of IL-6, IL-12, GM-CSF, KC, MCP-1, MIP-α, TNF-α	
CuONPs	Neutrophils	-Reduced bacterial killing	[44–46]
Fe_2_O_3_NPs	Lung lymph node cells	-Increased cytokine production	[47]
ZnONPs	Monocyte/Macrophages	-Toxicity (dose-dependent)	[48]
		-Cell death (ion relese-dependent)	
CoNP	Human PBMC	-Autophagy	[20]
PdNPs	Human PBMC	-Autophagy	[20]
		-Cell cycle (prolongation of G1-phase)	
		-Increase of IL-8	
Coated NPs	Monocyte-derived macrophages	-Variable cytotoxicity	[62–64, 69, 70]

### Immune stimulation

There are no evidences so far that NPs are able to induce a T- and B-cells (antibody) mediated specific immune reactions. In addition, there are no reports on IgE mediated allergies against NPs. However, three studies demonstrated the generation of particle-specific antibodies when C60 fullerene derivatives conjugated to a protein carrier were used for immunization [71–73]. All the studies demonstrating the generation of antibodies against NPs have as common feature the NPs conjugation to BSA. Therefore, it may be assumed that some water-soluble NPs may behave as haptens, gaining antigenicity when bind to protein carrier possibly as a result of their small size. On the contrary, other studies using gold colloids, different fullerene derivatives, and dendrimers, even in the presence of strong adjuvants, have not reported particle-specific immune response [74–76]. However, NP interaction with the innate immune system can influence the adaptive immune reaction through the production of cytokines and chemokines. AgNPs-exposed peripheral blood mononuclear cells produced IL-1β, a critical cytokine involved in lymphocyte activation and proliferation [39]. Also in this case, the size of NPs (5 nm and 28 nm) is inversely correlated to the magnitude of the observed effects. Both particle sizes induced inflammasome formation and the subsequent caspase-1 activation, but the 5 nm AgNPs produced more hydrogen peroxide and were more cytotoxic [39].

The interaction of NPs with the innate and adaptive immune system is modulated through the induction of specific pattern of cytokines. By this way, MeNPs can favor sensitization to common allergens. For instance, in mice exposed to TiO_2_NPs it was observed lung cellular inflammation involving eosinophils [77], with consequent production of T helper cells-activating cytokines [78] along with an amplification of Th2 cytokines expression. This event might contribute to the immunotoxicity underlying pulmonary injury associated with exposure to this type of NPs [79].

Similar pattern of Th2 cytokines production is induced by other MeNPs for example Ag- and ZnONPs [79–81]. On the contrary, Fe_2_O_3_-, NiO-, Co_3_O_4_- and PdNPs favor the production of Th1 cytokines by peripheral monocytes [24, 41, 82–84] hypothetically favoring the spreading of autoimmune diseases. Table 3 summarizes the immunostimulating effects of MeNPs.

**Table 3:** Immunostimulating effects of metal nanoparticles on cells of the immune system

NPs	Cells	Effects	Ref
AgNPs	Human PBMCs	Size dependent IL-1β production (inverse correlation)	[39]
TiO_2_NPs AgNPs ZnONPs	Lung macrophages, dendritic cells, basophils, neutrophils and eosinophils	Increase of inflammatory and Th cell activating cytokines (IL-2, IL-4, IL-6, CINC-1, IL-10, TNF-α)	[77–79, 86, 87]
Fe_2_O_3_ NiNP CoNPs PdNPs	Human peripheral monocytes	Increase of Th1 cytokines	[24, 41, 82–84]

### Adjuvant properties of MeNPs

The ability to stimulate the innate immune system is exploited in medicine as some NPs can guide appropriate immune responses in therapeutic settings acting as adjuvants [85]. In fact, in the immunotherapy of allergy, specific NPs imprint differentially modulated induction of acute allergic airway inflammation, with a significant inhibition of adaptive allergen-specific immunity. In this context, NPs are taken up by a specific subset of lung APC, stimulate cytokine/chemokine production and pulmonary DC maturation and translocate to the lung-draining lymph nodes via cell-associated transport. These findings support the development of lung-specific particulate vaccines, drug delivery systems, and immunomodulators [86]. The increase in inflammatory cells, airway hyperresponsiveness, increased levels of IL-4, IL-5, and IL-13, and the increased NF-κB levels in lungs after ovalbumin inhalation were significantly reduced by the administration of AgNPs. These are able to reduce intracellular ROS levels in bronchoalveolar lavage fluid induced by antigen inhalation [87]. Fullerenes have shown activity as a negative regulator of allergic inflammation, suppressing Ag-driven mediator release by mast cells [88]. Human mast cells preincubated with fullerenes exhibited a significant inhibition of IgE dependent mediator release, involving profound reductions in the activation of signaling molecules, likely involving a reduction in the tyrosine phosphorylation of Syk. In addition, fullerenes significantly inhibited elevation in cytoplasmic ROS levels induced by allergens [88]. Au- and AgNPs of similar size are taken up in a dose-dependent manner (more efficiently when they own a positive charge) by mouse peritoneal mast cells, whose efficiency of the degranulation and secretion was inhibited [89]. AuNPs of 15 nm are also successfully used as adjuvants of a recombinant protein vaccine (hNgR-Fc) developed to block myelin associated inhibitors of neurite outgrowth. In a rat model of spinal cord-injury, adjuvant AuNPs produced higher titers of anti-NgR antibody and promoted repair [90].

Aluminum is included in vaccines formulations, including those for allergen immunotherapy, for its adjuvant activity involving the engagement of the NLRP3 inflammasome and the induction of IL-1β by dendritic cells [91].

Aluminum has been designed in form of NPs (nanorods) to obtain effective immune adjuvancy using aluminum oxyhydroxide (AlOOH-). In in vitro models (human myeloid cells and murine dendritic cells), the adjuvant capacity of these NPs has been confirmed since they induce activation of the inflammasome. The extent of the activation depends on the shape, crystallinity and hydroxyl groups displayed of the NPs surface and is more potent than the elemental aluminum. Moreover, AlOOH-nanorods of specific shape and crystalline structures are capable of inducing higher MHC-II and co-stimulatory molecules expression [92]. Thus, they are useful for quantitative boosting of antigen-specific immune responses.

Superparamagnetic Fe_2_O_3_NPs, tested as an anti-cancer DC-targeting nanovaccine, were found to rapidly enter, even if transitorily, within endolysosomal compartments of ex vivo exposed dendritic cells, and also limitedly in the cytoplasm [93]. Magnetic Fe_2_O_3_NPs induced exosomes in the alveolar region of BALB/c mice that act as signaling mediators in the induction of Th1 immune activation. NPs induced exosomes would transfer their membrane-bound antigens to immature DC and macrophages, favoring their maturation into cells producing the Th1 cytokines, IL-12 and TNF-α which drive T-cell activation and differentiation [82]. Th1-polarized immune activation can be useful in the case of tumor nanovaccines. Table 4 summarizes the adjuvant properties of MeNPs.

**Table 4:** Immune adjuvant properties of metal nanoparticles

NPs	Cells	Effects	Ref
AgNPs	Lung	-Reduction of IL-4, IL-5, IL-13	[87]
		-Reduction of NF-kB	
		-Reduction of eosinophilic inflammation (OVA-induced )	
Fullerenes	Mast cells	-Inhibition of IgE dependent mediator release	[88]
		-Reduction of phosphorylation Syk	
		-Inhibition of allergen-induced ROS	
AuNPs AgNPs	Mast cells	-Inhibition of degranulation and serotonine secretion	[89]
AgNPs	Rat model of spinal cord injury	-Higher anti-NgR antibody	[90]
AlNPs	Dendritic cells	-Increased MHC and CD86, Cd80 and CD40 (> than Al ions)	[91]
Fe_2_O_3_NPs	Dendritic cells	-Th1 immune activation	[81, 93]

### NPs and physical barriers of the innate immune system

Since epithelial cells have toll-like receptors [94] and secrete cytokines [95] that participate in the determination of the type of immune reaction against the host, it is important to verify the ability of NPs to overcome the epithelial barrier and to look at the possible toxic effects on the epithelial cells. Inhalation is an important route for NPs exposure, and several studies report NPs-induced lung inflammation in animals [96, 97], although no examples of lung pathologies have been reported in humans. The production of ROS remains the main inflammatory mechanism induced by Ag-, TiO_2_-, ZnO-, MnO_2-_ and CeO_2_NPs in human bronchial epithelium [98–104]. On the contrary, oxidative stress plays only a marginal role in the genotoxicity of Fe_2_O_3_NPs in human lung cells [105]. ROS production can lead to cellular and DNA damage [106] with extracellular and intracellular signals and cell death through apoptosis [107] that has been associated with many lung diseases [108]. A kinetic study of the gene expression profiles induced by inhalation of Co_3_O_4_ and CeO_2_ NPs in lung epithelial cells showed mainly a down-regulation of gene transcription; about 14 % of the differentially expressed transcripts were involved in immune processes [109]. NiNPs induced a significant reduction of cell viability and an increase of apoptotic and necrotic cells at 24 h along with an increase in ROS production and a significant release of IL-6 and −8, dependent on mitogen activated protein kinases (MAPK) cascade through the induction of NF-kB pathway [110]. ZnO NP exposed human bronchial epithelial cells significantly increase the expression of IL-8 mRNA and protein in a dose-dependent manner [111]. Both IL-6 and IL-8 are proteins of the acute of the acute inflammation acting as chemotactic and activating factors for neutrophils and other granulocytes [13].

Generation of ROS and release of IL-8 are also typical for MeNPs exposed gastrointestinal epithelial cells. ZnONPs induce cell oxidative damage, the small-sized NPs being the more effective, with a marked increase in anti-oxidant gene expression and high lipid peroxidation level in the enterocytes, in which disarrangement of the cytoskeleton and cell junction integrity were evidenced. These events led to diffuse necrotic damages in the intestinal barrier with a trans- and paracellular permeation of NPs through the mucosa. Differently from other NPs, ZnONPs toxicity seems to be crucially mediated by the NP reactivity rather than their dissolved ions [112]. ZnONPs led to significant cell death in Caco-2 and SW480 cells, while Ag and TiO_2_NPs led to cell death in SW480 cells. In these last NPs, the exposure did not yield significant increased ROS generation, but all NP exposures led to increased IL-8 cytokine generation in both cell lines [113].

Mucus represents an efficient acellular barrier for both the respiratory and intestinal tracts. The passage through the mucus is most likely based on the electrostatic repulsion from negatively charged sugar moieties which favors the penetration of positively charged hydrophilic molecules. The passage of lipophilic compounds is slow [114]. Furthermore, smaller particles underwent a significantly faster transport so bypassing the barrier.

It has been demonstrated that, *in vitro*, TiO_2_NPs [115] and, *in vivo*, AgNPs [116] induce abnormal mucus production. However, no data are available on the capacity of NPs to pass the mucosal layer of bronchi and intestine in pathological conditions.

Once reached the intestinal epithelium, NPs can react with epithelial cells. It was found that AgNPs damage microvilli as well as intestinal glands in mice, inducing a malabsorption syndrome [117]. Au NPs were retained in the gut lumen of *Daphnia magna*, but there was no observable internalization into the gut epithelial cells. Carbon nanotubes and CuO NPs have a similar behavior as *in vivo* retention does not necessarily result in their internalization [118].

TiO_2_NPs can cross the intestine. In fact, exposure of whole gut sacs to 1 mg/l TiO_2_NPs for 4 h caused total Ti metal concentrations to increase in the intestine in rainbow trout [119]. Furthermore, TiO_2_NPs cross Caco-2 monolayers without disruption of junctional complexes and without causing cytotoxicity [120]. Since the plasma membrane of the cells forming the epithelial barrier is lipophilic, lipophilic substances are taken up passively by the transcellular route whereas hydrophilic drug compounds use the paracellular route. The penetration area of the paracellular route is extremely small compared to the transcellular route and restricted to polar substances below 1000 Da. NPs are not expected to be able to use the paracellular route, because they are considerably larger than 1000 Da. Transcellular passage by passive diffusion appears to be rare. Although the passage of cells by 22 nm TiO_2_ NPs was suggested to occur by passive diffusion [121], 5–8 nm AuNPs could not enter cells by this process [121]. Independently on the entry route, NPs are mainly transported via endosomes to lysosomes. Non-functionalized Ag-, TiO_2_- and SiO_2_NPs are mainly taken up by clathrin-mediated endocytosis [122–124].

The transport of antigens and/or NPs is mainly carried out by the follicle associated epithelium-M cells, since the mucus layer limits the particle uptake across the villous epithelium [125]. Once NPs have crossed the epithelial barrier, they can be found in the lymphatic tissues.

No information is present in literature regarding possible changes in MeNPs uptake in inflamed intestine, whereas contrasting results have been obtained with other NPs [126, 127]. Other important effects are linked to the tendency of NPs to absorb macromolecules. By adsorption of organic compounds also unintended molecules (undigested and unmetabolized compounds) may be absorbed by the gastrointestinal tract so inducing adverse effects [128]. Table 5 summarizes the interaction of MeNPs with cellular barriers of the innate immunity.

**Table 5:** Nanoparticle interaction with cell barriers of the immune system

NPs	Cells	Effects	Ref
AgNPS TiO_2_NPs ZnONPs MnONPs CeO_2_NPs	Human lung epithelium	-ROS production	[97–104]
		-DNA damage	
		-Apoptosis	
Fe_2_O_3_NPs	Human bronchial epithelium	-Genotoxicity	[105]
NiNPs	Lung	-Reduction of cell viability	[110]
		-Apoptosis	
		-Necrosis	
		-Increase of ROS, IL-6 and IL-8 (MAPK-dependent, NF-kB-mediated)	
ZnNPs	Human bronchial epithelial cells	-Increased of IL-8 (dose-dependent)	[111]
		-Activation of NFκB and C/EBPβ	
MeNPs ZnONPs	Gut epithelium	-Generation of ROS	[112]
		-Release of IL-8	
		-Cytotoxicity (size dependent)	
AgNPs TiO_2_NPs	Intestinal epithelium	-Cell death (SW480 cells)	[113]
		-IL-8 production	
		-ROS generation	
AgNPs	Mice intestinal epithelium	-Damage of microvilli and intestinal glands	[117]
		-Malabsorption syndrome	
TiO_2_NPs	Caco-2	-Cross monolayers without disruption of junctional complexes and without causing cytotoxicity	[120]
TiO_2_NPs AgNPs	Bronchial and intestinal eptithelium	-Abnormal mucus production	[115, 116]

## Conclusion

The study of the possible effects of potentially noxious substances on the innate immune system is of fundamental importance, as it drives the subsequent reaction by the adaptive immune system. Cells of the innate immune system acting as APC are responsible of antigen recognition. Damaged APC can release pattern of cytokines that can start hypersensitive immune reactions with the appearance of allergies of autoimmune diseases and in industrialized countries hypersensitivity reactions represent the most frequently reported immunotoxic effects of chemicals [129, 130].

Moreover, the innate immune system can directly react against harmful substances thus inducing inflammation, which is the basis of many diseases involving different organs. Among them cancer is the main concern as immunomodulation also plays a key role in carcinogenicity. Immunosuppressive activity is important as neoplastic cells frequently have antigenic properties that permit their detection and elimination by normal immune system function. It has been demonstrated that, if a compound is immunotoxic, likely it is carcinogenic [131]. Chemicals affecting the activity of NK cells, NKT cells, macrophages, CD8^+^ cytotoxic T lymphocytes, or altering cytokine production, are likely to compromise cancer immune surveillance.

Based on the *in vitro*, *in vivo* and occupational data, it can be expected that Me NPs may activate the immune system towards immune suppression and immune activation in exposed people. In fact, Me NPs induce cell death of immune cells and changes in cytokine production. Therefore, this tends to support the hypothesis that Me NPs directly or indirectly interacting with cells of the immune system trigger effects that may be relevant to the development of escalating diseases (allergies, autoimmunity and cancer) in Western countries.

On the contrary, literature review clearly shows that there are no report of immune diseases induced by MeNPs exposure, except for the observation that the increase in Pd allergic contact dermatitis is linked to the increased exposure to PdNPs released from car catalytic converters [58]. Descotes [132] stated that “present methods of evaluating immunotoxicity are primarily focused on immunosuppression, even though unexpected immunosuppression has rarely a cause of concern”. On the other hand, immune system has enormous overcapacities and indeed a functional deficit often manifests itself only under the additional stress of mass infection. Even a therapeutic immunosuppression is difficult to reach; in fact, it needs strong doses, strict adherence to the dose regimen, and often a combination of drugs. It is also important to underline that immune responses in the normal human population vary considerably. This means that immunomodulation does not necessarily take an individual out of a healthy response pattern.

A few studies undertaken by groups without a specialist toxicological qualification generated results that are not based on scientific fundamentals and speak of the “enormous toxicological potential” of engineered NPs. These statements have a negative effect on the public opinion much greater than the many good scientific studies which demonstrate, through careful analysis of the dose–response relationship, that we are operating in a safe area.

It is clear from the literature that the influence MeNPs have in the innate immune system could be beneficial when exploited to modify the immune response in medicine. In fact, recent MeNPs able to stimulate of immune functions are used in the development of new vaccines, to promote immunity against tumors and suppress autoimmunity. Therefore, understanding the modality of the effect of Me NPs on immune responses is an essential requirement to developing novel technological and clinical applications. Understanding the immune compatibility of nanoformulations is one of the important factors in (pre)clinical development and requires reliable *in vitro* and *in vivo* immunotoxicity tests. There are several major challenges in the *in vitro* testing of nanoparticle immunotoxicity: i) selection of a model, ii) selection of an end-point, iii) selection of relevant positive and negative controls, iv) nanoparticle interference with in vitro assays, and v) understanding assay predictability of corresponding immunotoxicity *in vivo*. The generally low sensitivity of standard *in vivo* toxicity tests to immunotoxicities, inter-species variability in the structure and function of the immune system, high costs and relatively low throughput of *in vivo* tests, and ethical concerns about animal use underscore the need for trustworthy *in vitro* assays.

In conclusion, MeNPs represent a technological advancement that may also help the development of new and more potent therapeutic tools. However, in this review we show that their non-intentional exposure might cause, at least theoretically, effects on the immune system.

This fact represents a paradox that toxicologists and developers have to overcome by the production and the dispersion of nanoparticles in work and living environments, and their entry in the food chain. This is a major issue that makes these technologies to be improved from the point of view of minimizing the associated risk. Assessment of bioavailability and exposure of workers and general population appear to be a must for a successful and safe forthcoming development and application of nanotechnology.
